# Supplementary material for: Neuropeptides NLP-10 and FLP-18 stimulate lipid catabolism in chronically diet-restricted Caenorhabditis elegans
Source: bioRxiv. 2026 Sep 23:2026.09.17.752367. Preprint. [Version 1] doi: 10.64898/2026.09.17.752367 (PMC13622093; doi:10.64898/2026.09.17.752367)
Supplement: Supplementary file 1 [file NIHPP2026.09.17.752367v1-supplement-1.pdf]

bioRxiv preprint doi: https://doi.org/10.64898/2026.09.17.752367; this version posted September 23, 2026. The copyright holder for this preprint (which was not certified by peer review) is the author/funder, who has granted bioRxiv a license to display the preprint in perpetuity. It is made available under a CC-BY 4.0 International license.

SUPPLEMENTARY TABLES

Supplementary Table1. Relative gene expression levels by qRT-PCR

Supplementary Table2. Intestinal fat levels of animals with BMP deficiency with or without functional neuropeptides

Supplementary Table 3. TG-to-DG ratio of wildtype and *sma-6(wk7)* calculated by the sum of area-under-curve values in LC-MS

Supplementary Table 4. Reagent and resource used in this study

Supplementary Table 5. Oligos used in this study

Supplementary Table 6. Raw data for figure plotting
